# Shikonin Attenuates Concanavalin A-Induced Acute Liver Injury in Mice via Inhibition of the JNK Pathway

**DOI:** 10.1155/2016/2748367

**Published:** 2016-05-16

**Authors:** Tong Liu, Yujing Xia, Jingjing Li, Sainan Li, Jiao Feng, Liwei Wu, Rong Zhang, Shizan Xu, Keran Cheng, Yuqing Zhou, Shunfeng Zhou, Weiqi Dai, Kan Chen, Fan Wang, Jie Lu, Yingqun Zhou, Chuanyong Guo

**Affiliations:** ^1^Department of Gastroenterology, Shanghai Tenth People's Hospital, Tongji University School of Medicine, Shanghai 200072, China; ^2^The First Clinical Medical College, Nanjing Medical University, Nanjing 210029, China; ^3^The First Affiliated Hospital of Soochow University, Suzhou 215006, China

## Abstract

*Objective*. Shikonin possesses anti-inflammatory effects. However, its function in concanavalin A-induced acute liver injury remains uncertain. The aim of the present study was to investigate the functions of shikonin and its mechanism of protection on ConA-induced acute liver injury.* Materials and Methods*. Balb/C mice were exposed to ConA (20 mg/kg) via tail vein injection to establish acute liver injury; shikonin (7.5 mg/kg and 12.5 mg/kg) was intraperitoneally administered 2 h before the ConA injection. The serum liver enzyme levels and the inflammatory cytokine levels were determined at 3, 6, and 24 h after ConA injection.* Results*. After the injection of ConA, inflammatory cytokines IL-1*β*, TNF-*α*, and IFN-*γ* were significantly increased. Shikonin significantly ameliorated liver injury and histopathological changes and suppressed the release of inflammatory cytokines. The expressions of Bcl-2 and Bax were markedly affected by shikonin pretreatment. LC3, Beclin-1, and p-JNK expression levels were decreased in the shikonin-pretreated groups compared with the ConA-treated groups. Shikonin attenuated ConA-induced liver injury by reducing apoptosis and autophagy through the inhibition of the JNK pathway.* Conclusion*. Our results indicated that shikonin pretreatment attenuates ConA-induced acute liver injury by inhibiting apoptosis and autophagy through the suppression of the JNK pathway.

## 1. Introduction

Hepatitis is caused by many factors including alcohol, viruses, drugs, immune injury, and idiopathic factors and has remained a serious human health problem throughout the world [1, 2]. The features of hepatitis are the release of inflammatory cytokines, the elevation of aspartate aminotransferase (AST) and alanine aminotransferase (ALT), and hepatocyte apoptosis and necrosis. Although many treatments are currently used in the clinic, the therapeutic effect is not ideal. Therefore, effective treatment measures need to be explored.

Concanavalin A (ConA) has the ability to stimulate the activation of T lymphocytes, mostly CD4^+^ T-cells, leading to immune hepatitis in mice [3, 4]. Natural killer (NK) T-cells and macrophages are also closely associated with the progression of liver injury caused by ConA [5]. Therefore, ConA-induced hepatitis is an ideal model to investigate the mechanisms and treatments of T-cell-dependent hepatotoxicity. Injection of ConA increases the proinflammatory cytokines IL-1*β*, IL-6, IFN-*γ*, and TNF-*α* [6–9]. Research has shown that there is a close relationship between high cytokine levels and early liver injury [10]. Moreover, IL-1*β* is a central cytokine associated with liver injury [11, 12].

Several signaling pathways have been associated with the underlying mechanisms of ConA-induced hepatitis. Studies have shown that ConA-induced immune hepatitis was significantly attenuated with the inhibition of the phosphorylation of JNK [13–16]. Apoptosis or programmed cell death is associated with liver injury caused by ConA [15, 17]. Bcl-2 family members, including Bcl-2, Bcl-xl, Bax, and Bad, play key roles in the apoptotic pathway. Bax and Bad represent proapoptotic proteins, while Bcl-2 and Bcl-xl represent antiapoptotic proteins. A proper balance between Bcl-2 and Bax determines cell survival and cell death.

Autophagy, first described by Ashford and Porter, is characterized by the formation of autophagosomes and autolysosomes and is an intracellular degradation system that focuses on defective organelles [18]. Autophagy plays important roles in various biological processes, including innate immunity, inflammatory responses, and adaptive immunity [19]. However, autophagy is also called type II programmed cell death and is intimately associated with eukaryotic cell death and apoptosis. Therefore, we consider that autophagy is a double-edged sword. Recent studies show that autophagy is linked with negative regulatory mechanisms in the liver. Microtubule-associated protein 1 light chain 3 (LC3) and Beclin-1 are widely considered as markers of autophagy [20].

Shikonin, a natural product extracted from* Lithospermum erythrorhizon*, has many biological functions, including antibacterial, antioxidant, anti-inflammatory, and antitumor activities [21]. Recently, shikonin was shown to play an important role in regulating the process of inflammation, exerting strong anti-inflammatory effects. Lee and colleagues found that shikonin could effectively inhibit allergic airway inflammation in a model of asthma and suppress bone marrow-derived dendritic cell (BM-DC) maturation in vitro [22]. Shikonin exerted anti-inflammatory effects by interfering with the degradation of I*κ*-B*α* and then suppressed the activation of NF-*κ*B, as described by Andújar and colleagues [23]. Xiong et al. also found that shikonin could reduce the release of proinflammatory cytokines in cerulein-induced acute pancreatitis in mice [24]. However, the mechanism of action of shikonin in a model of ConA-induced autoimmune hepatitis remains unclear.

The present study investigated the underlying mechanism of action of shikonin in ConA-induced autoimmune hepatitis. We hypothesized that shikonin could reduce the level of IL-1*β* upregulated in ConA-induced hepatitis and ameliorate liver injury, as measured by serum hepatic enzymes, proinflammatory cytokines, and histological changes, which may be partly associated with the C-Jun N-terminal kinase (JNK)/p-JNK pathway.

## 2. Materials and Methods

### 2.1. Reagents

Shikonin, dimethyl sulfoxide (DMSO), and ConA were purchased from Sigma-Aldrich (St. Louis, MO, USA). Antibodies used in the study were from Cell Signaling Technology (Danvers, MA, USA), including IL-1*β*, TNF-*α*, IFN-*γ*, LC3, Beclin-1, caspase 9, Bax, Bcl-2, total JNK, p-JNK, and P62. The PCR kit was purchased from Takara (Takara Biotechnology, Dalian, China). The microplate test kits for AST and AST were purchased from Nanjing Jiancheng Bioengineering Institute (Jiancheng Biotech, China).

### 2.2. Animals

Male Balb/c mice (6–8 weeks old, 23 ± 2 g) were purchased from Shanghai Laboratory Animal Co., Ltd. (Shanghai, China). The mice were housed in a clean environment at 24 ± 2°C and an alternating 12 h light and dark cycle. They were allowed free access to water and food. The study was approved by the Animal Care and Use Committee of Shanghai Tongji University.

### 2.3. Preliminary Study

A total of 48 mice were randomly divided into four groups as follows: a normal control group treated with saline solution, a DMSO group treated with 2% DMSO, and two shikonin groups treated with shikonin at doses of 7.5 mg/kg and 12.5 mg/kg. Shikonin was dissolved in 2% DMSO. Four mice were randomly selected and killed. The serum and liver tissues were gathered and used to analyze data immediately, including liver enzymes, the levels of cytokines, and pathological changes.

### 2.4. Drug Treatment

ConA was dissolved in normal saline solution at a concentration of 2.5 mg/mL and injected at 20 mg/kg via tail vein to induce acute hepatitis according to previous study [25]. Shikonin was diluted with 2% DMSO and injected intraperitoneally 2 h prior to ConA challenge. A total of 96 mice were treated by tail intravenous injection of ConA 2 h before administrating shikonin. The mice were randomly divided into four groups:

(1) Normal control (*n* = 24): mice were injected with saline solution only.
(2) ConA group (*n* = 24): mice were injected with 20 mg/kg ConA via the tail vein.
(3) Low dose group (*n* = 24): mice were intraperitoneally injected with 7.5 mg/kg shikonin 2 h before ConA challenge.
(4) High dose group (*n* = 24): mice were intraperitoneally injected with 12.5 mg/kg shikonin before ConA challenge.

### 2.5. Biochemical Analysis

Based on a previous study, blood was collected at three time points 3, 6, and 24 h rapidly after the mice were sacrificed. After blood collection, the serum was separated by centrifugation at 2000 rpm at 4°C for 10 min and used to detect liver function and cytokine levels. The levels of ALT and AST were measured with an automated chemical analyzer (Olympus AU1000, Japan). IL-1*β*, TNF-*α*, and IFN-*γ* were measured by enzyme-linked immunosorbent assay (ELISA) kits (R&D Systems, USA) according to the manufacturer's protocols.

### 2.6. Histopathology

The middle portion of the left liver lobe was cut and fixed in 4% paraformaldehyde for at least 24 h. After fixation, the specimen was embedded in paraffin; sections were cut at a thickness of 5 *μ*m and stained with hematoxylin and eosin (H&E). The inflammatory level and tissue damage were observed by light microscopy.

### 2.7. Immunohistochemistry

Prepared paraffin-embedded liver sections (5 *μ*m) were heated at 60°C for 1 h and then dewaxed and rehydrated by using xylene and different concentrations of alcohol. To recover the antigens, the paraffin-embedded sections were treated with an antigen-retrieval technique, including heating in a water bath at 95°C for 10 min, and then covered in hydrogen peroxide solution (3%) for 20 min at 37°C to block the activity of endogenous peroxidase. The nonspecific binding sites were blocked with 5% bovine serum albumin (BSA) at 37°C for 20 min and then incubated for 10 min at room temperature. The liver specimens were incubated overnight with IL-1*β* (1 : 100), TNF-*α* (1 : 100), IFN-*γ* (1 : 100), Bax (1 : 100), Bcl-2 (1 : 100), p-JNK (1 : 100), and LC3I/II (1 : 500). The next day, the liver sections were incubated with a secondary antibody, and a diaminobenzidine kit was used to analyze antibody binding. Finally, the slices were observed under a light microscope. The ratios of brown staining areas and total areas were calculated using Image-Pro Plus software 6.0.

### 2.8. Western Blotting

After recovery from −80°C storage, liver tissues were rapidly ground in liquid nitrogen and then lysed with RIPA lysis buffer supplemented with protease inhibitors (PI) and phenylmethanesulfonyl fluoride (PMSF). The protein concentration was detected with the bicinchoninic acid (BCA) protein assay (Kaiji, China). Equivalent amounts of total protein (120 *μ*g) were boiled and mixed with 5 × SDS-PAGE sample loading buffer. The proteins were separated by using different concentrations of sodium dodecyl sulfate (SDS) polyacrylamide gels and then transferred to polyvinylidene difluoride (PVDF) membranes. Nonspecific binding was blocked with 5% nonfat milk (diluted in PBS) for 1 h and incubated overnight at 4°C with primary antibodies: *β*-actin (1 : 1000), IL-1*β* (1 : 200), TNF-*α* (1 : 200), IFN-*γ* (1 : 200), Bcl-2 (1 : 500), Bax (1 : 500), caspase 9 (1 : 500), Beclin-1 (1 : 500), LC3 (1 : 1000), P62 (1 : 500), total JNK (1 : 1000), and p-JNK (1 : 500). Membranes were washed with PBST three times for 10 min and then incubated with a secondary goat anti-rabbit or anti-mouse antibody (1 : 2000) for 1 h at 37°C. Finally, the membranes were washed with PBST three times for 10 min and then scanned using the Odyssey two-color infrared laser imaging system.

### 2.9. Reverse Transcription- (RT-) PCR and Quantitative Real-Time- (qRT-) PCR

The liver tissue was detected and analyzed by qRT-PCR. Total RNA was extracted from frozen liver tissues and transcribed into cDNA using the reverse transcription kit (Takara Biotechnology, China), according to the manufacturer's protocols. SYBR Green Quantitative RT-PCR was performed to detect target gene expression using a 7900HT fast real-time PCR system (Applied Biosystems, CA, USA), according to the protocols for SYBR Premix EX Taq (Takara Biotechnology (Dalian) Co., Ltd., Dalian, China). The primers used in the study are listed in Table 1.

### 2.10. ROS Assay

The fresh liver tissues were fixed in 4% paraformaldehyde for 1 h. Then the fixed liver tissues were washed three times by PBS for 15 min before they were dehydrated overnight in 30% sucrose at 4°C. Then the sections were infiltrated with OCT (Sakura, USA) for 2 h and stored at −80°C. 5 *μ*m liver sections were cut by a freezing microtome. The prepared sections (5 *μ*m) dried at room temperature for 10 min; then the sections were washed three times by PBS for 5 min each time. And then the prepared sections were incubated with ROS Fluorescent Probe-DHE (60 *μ*M, diluted by PBS) for 90 min. After incubation, the sections were washed three times by PBS for 5 min each time. The sections were enclosed with quenching agent and acquired with fluorescence microscopy. The whole process needs to avoid light.

### 2.11. Statistical Analysis

All the experiments were repeated at least three times. The results are expressed as the mean ± SD. One-way analysis of variance (ANOVA) was used to analyze the datum of q-PCR, ELISA, ROS scavenging activity, the levels of AST and ALT, the gray value of western blotting, and the areas of inflammation and necrosis in immunohistochemistry. In all comparisons, *P* values < 0.05 were considered statistically significant. All statistical analyses were performed using GraphPad Prism (v 6.0) software.

## 3. Results

### 3.1. 2% DMSO and Shikonin Had No Effect on Liver Function or the Inflammatory Response

**Figure 1:**
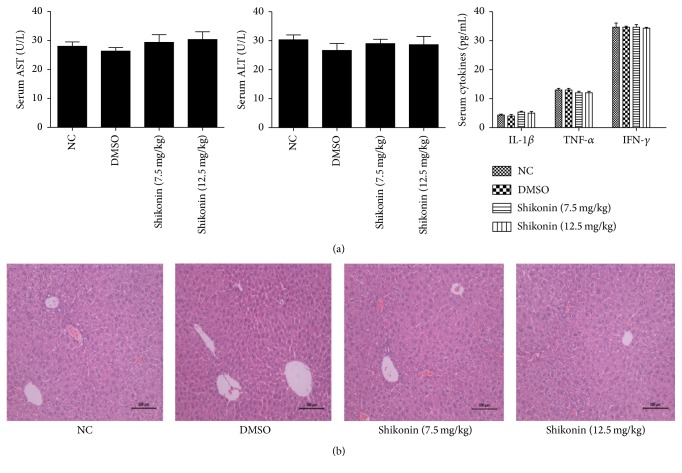
Effects of 2% DMSO and shikonin on the liver function and pathology of healthy mice. (a) The levels of serum ALT and AST in the four groups did not differ. Data are given as means ± SD (*n* = 4, *P* > 0.05). The serum levels of TNF-*α*, IL-1*β*, and IFN-*γ* of four groups were evaluated in each group with ELISAs (*n* = 4, *P* > 0.05). (b) Representative hematoxylin-and-eosin-stained sections of the liver. Original magnification, ×200.

To determine whether the drug and the solvent affected liver function, we examined the effects of shikonin and 2% DMSO on liver enzymes and the release of cytokines. As shown in Figure 1(a), serum AST and ALT levels did not differ between the 2% DMSO and shikonin groups and the control group. The serum IL-1*β*, TNF-*α*, and IFN-*γ* levels were comparable between the four groups.  Figure 1(b) shows no distinct necrosis in the four H&E stained images.

### 3.2. Pretreatment with Shikonin Ameliorated ConA-Induced Acute Hepatitis in Mice

**Figure 2:**
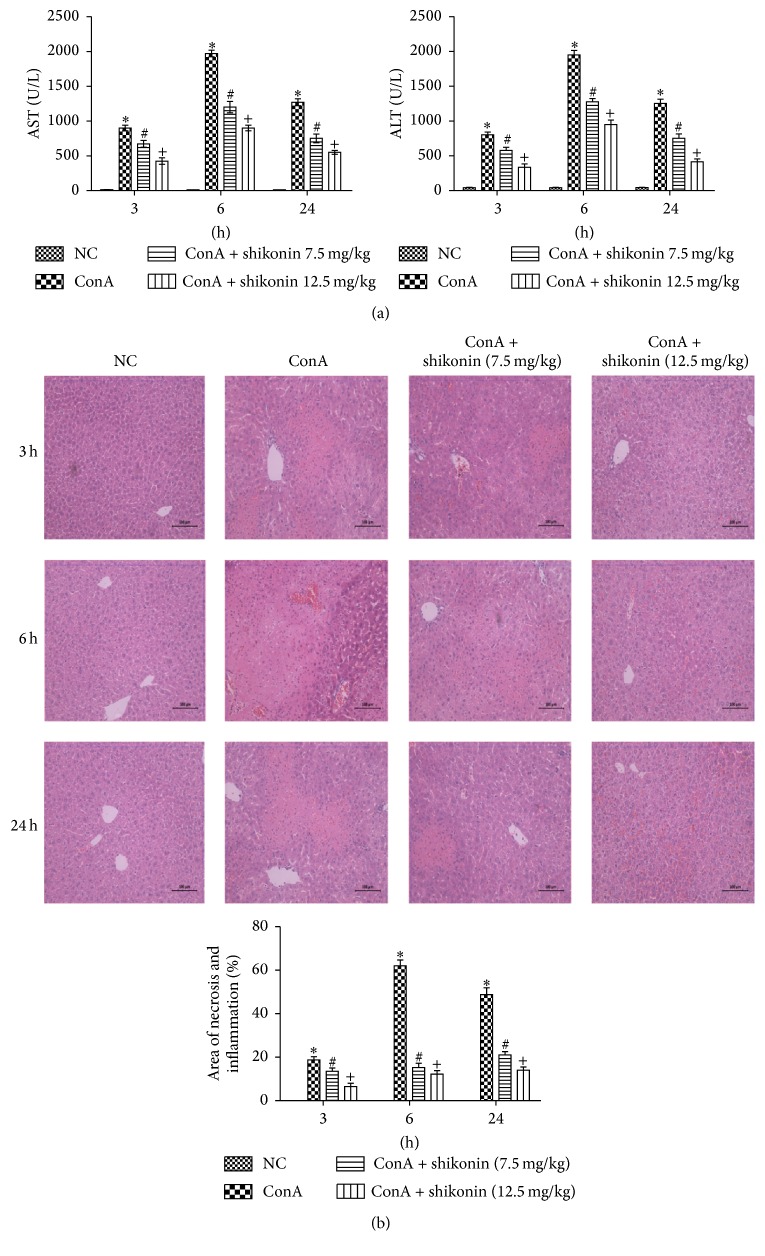
Effects of shikonin on liver function and pathology of mice with ConA-induced acute hepatitis. (a) The levels of serum ALT and AST changed depending on the shikonin dose, 7.5 mg/kg or 12.5 mg/kg. Data are given as means ± SD (*n* = 8, ^*∗*^ *P* < 0.05 for NC versus ConA, ^#^ *P* < 0.05 for ConA + shikonin (7.5) versus ConA, and ^+^ *P* < 0.05 for ConA + shikonin (12.5) versus ConA). (b) The necrotic and edematous area stained with hematoxylin and eosin and used for the liver sections was analyzed with Image-Pro Plus 6.0 (magnification, ×200). The results show statistically significant differences among the different groups (*n* = 8, ^*∗*^ *P* < 0.05 for NC versus ConA, ^#^ *P* < 0.05 for ConA + shikonin (7.5) versus ConA, and ^+^ *P* < 0.05 for ConA + shikonin (12.5) versus ConA).

ConA can activate T-cells to induce autoimmune hepatitis. To detect the effect of shikonin on ConA-induced hepatitis, mice were pretreated with shikonin 2 h before ConA injection. Based on the experimental design, the serum and liver tissues of mice were collected at three time points: 3, 6, and 24 h.  Figure 2(a) shows that the serum AST and ALT levels were significantly increased at the three time points, with a peak at 6 h after ConA injection. However, pretreatment with shikonin clearly reduced the serum levels of liver enzymes and the high dose was more effective. The same result was detected in the histopathological study. As shown in Figure 2(b), abundant necrotic areas were observed in the ConA group, while the shikonin groups showed minor liver injury at the three time points, indicating that pretreatment with shikonin dramatically decreased liver necrosis. The changes were more marked in the high dose group. Image-Pro Plus software showed statistically significant differences between the groups. Taken together, these results indicated that pretreatment with shikonin effectively reduced ConA-induced liver injury in mice.

### 3.3. Shikonin Pretreatment Decreased Inflammatory Responses in ConA-Induced Hepatitis

**Figure 3:**
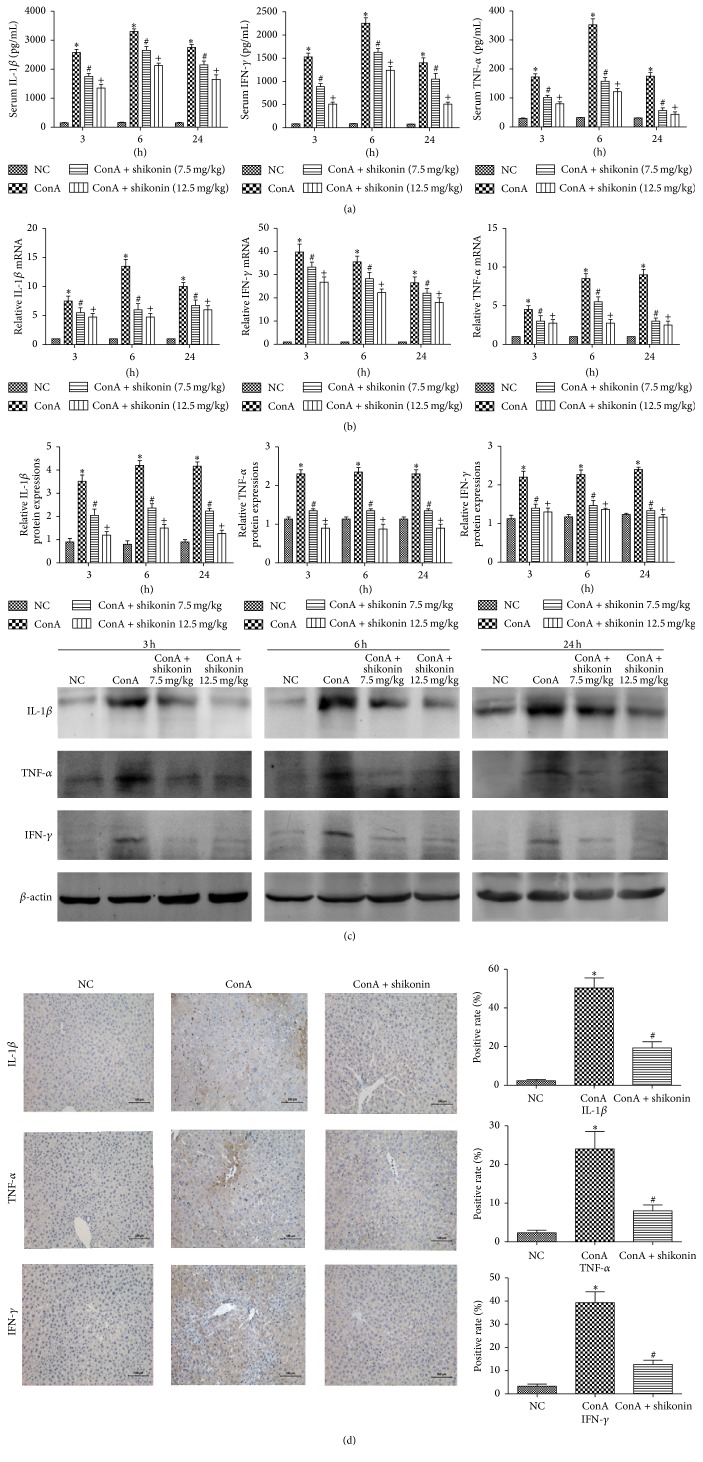
Effects of shikonin on the production of IL-1*β*, TNF-*α*, and IFN-*γ* in mice with ConA-induced acute hepatitis. (a) The levels of serum TNF-*α*, IL-1*β*, and IFN-*γ*, measured with ELISAs were reduced by shikonin pretreatment in mice at doses of both 7.5 mg/kg and 12.5 mg/kg. Data are presented as means ± SD (*n* = 8, ^*∗*^ *P* < 0.05 for NC versus ConA, ^#^ *P* < 0.05 for ConA + shikonin (7.5) versus ConA, and ^+^ *P* < 0.05 for ConA + shikonin (12.5) versus ConA). (b) The mRNA levels of IL-1*β*, TNF-*α*, and IFN-*γ* were evaluated in each group with real-time PCR (*n* = 8, ^*∗*^ *P* < 0.05 for NC versus ConA, ^#^ *P* < 0.05 for ConA + shikonin (7.5) versus ConA, and ^+^ *P* < 0.05 for ConA + shikonin (12.5) versus ConA). (c) The expression levels of the IL-1*β*, TNF-*α*, and IFN-*γ* proteins were determined with western blotting and the gray values were calculated (*n* = 8, ^*∗*^ *P* < 0.05 for NC versus ConA, ^#^ *P* < 0.05 for ConA + shikonin (7.5) versus ConA, and ^+^ *P* < 0.05 for ConA + shikonin (12.5) versus ConA). (d) Immunohistochemistry staining (×200) showing the expression of IL-1*β*, TNF-*α*, and IFN-*γ* in liver tissue at 6 h. The ratio of brown area to total area was analyzed with Image-Pro Plus 6.0 (*n* = 8, ^*∗*^ *P* < 0.05 for NC versus ConA, ^#^ *P* < 0.05 for ConA versus ConA + shikonin).

The development of autoimmune hepatitis is closely associated with the release of proinflammatory cytokines, including IL-1*β*, TNF-*α*, and IFN-*γ*. The levels of IL-1*β*, TNF-*α*, and IFN-*γ* were determined by ELISA and found to be significantly increased in the ConA group, showing a peak at 6 h after ConA induction (Figure 3(a)). Shikonin pretreatment dramatically reduced the levels of these proinflammatory cytokines, particularly at 6 h. To confirm our results, we used real-time PCR to detect the mRNA expression of IL-1*β*, TNF-*α*, and IFN-*γ* at each time point. The mRNA expression of these cytokines was decreased by shikonin pretreatment compared with the ConA group, especially in the high dose group (Figure 3(b)). Western blot analysis was performed to determine the protein expression of these cytokines. The protein expression of IL-1*β*, TNF-*α*, and IFN-*γ* was significantly increased in the ConA group at all the three time points and reached peak at 6 h (Figure 3(c)), consistent with the mRNA expression. However, the protein expression of these cytokines decreased in both shikonin pretreatment groups, and shikonin administered at 12.5 mg/kg was more effective, indicating that the effects of shikonin on ConA-induced hepatitis were dose-dependent. Immunohistochemical staining was used to determine the expression of the inflammatory cytokines in the normal control, ConA, and ConA + shikonin groups (Figure 3(d)). These results provided strong evidence that pretreatment with shikonin could decrease the release of inflammatory cytokines such as IL-1*β*, TNF-*α*, and IFN-*γ* in autoimmune hepatitis caused by ConA.

### 3.4. Shikonin Attenuated Hepatocyte Apoptosis and Autophagy in ConA-Induced Autoimmune Hepatitis

**Figure 4:**
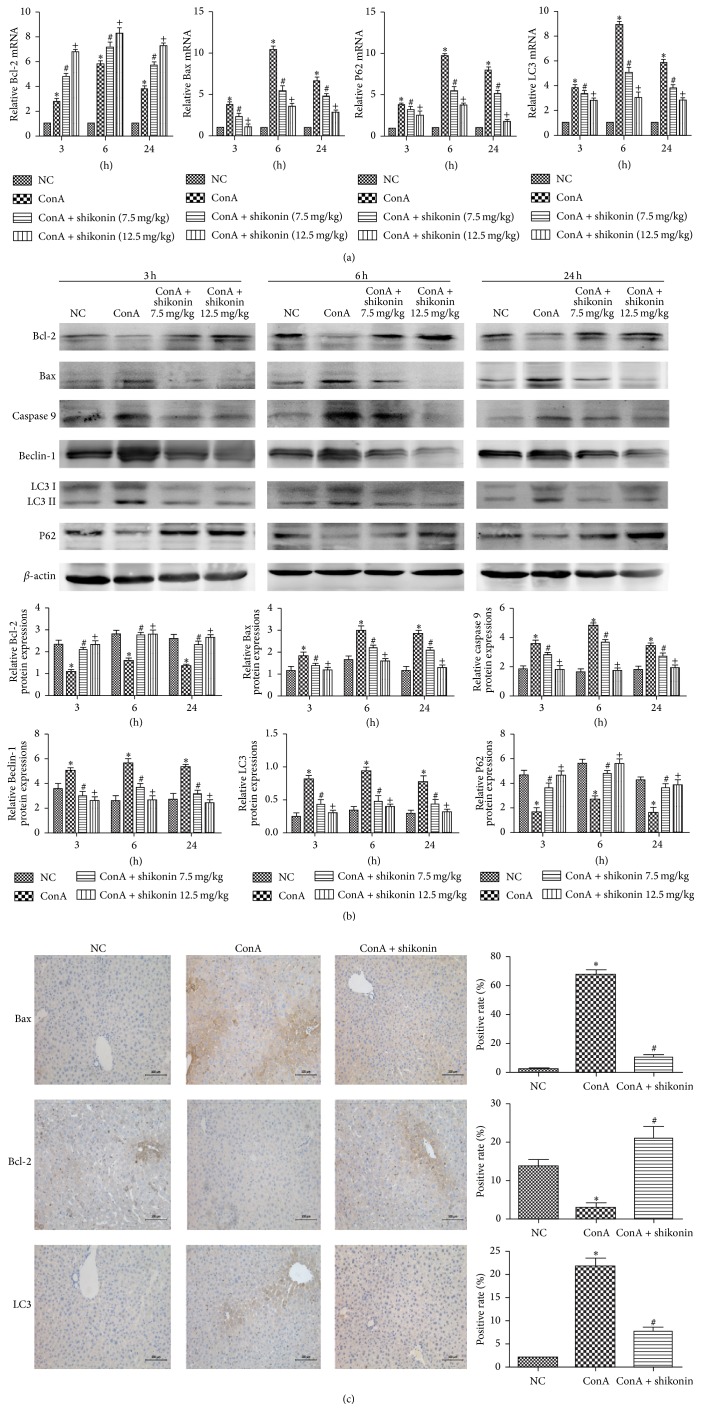
Effects of shikonin on apoptosis and autophagy in mice with ConA-induced acute hepatitis. (a) cDNA levels of LC3, P62, Bcl-2, and Bax were measured with real-time PCR (*n* = 8, ^*∗*^ *P* < 0.05 for NC versus ConA, ^#^ *P* < 0.05 for ConA + shikonin (7.5) versus ConA, and ^+^ *P* < 0.05 for ConA + shikonin (12.5) versus ConA). (b) Protein expression of LC3, Beclin-1, P62, Bcl-2, Bax, and caspase 9 was detected with western blotting and the gray values were calculated (*n* = 8, ^*∗*^ *P* < 0.05 for NC versus ConA, ^#^ *P* < 0.05 for ConA + shikonin (7.5) versus ConA, and ^+^ *P* < 0.05 for ConA + shikonin (12.5) versus ConA). (c) Immunohistochemistry staining (×200) showed the expression of Bcl-2, Bax, and LC3 protein in liver tissue at 6 h. The ratio of brown area to total area was analyzed with Image-Pro Plus (v 6.0) (*n* = 8, ^*∗*^ *P* < 0.05 for NC versus ConA, ^#^ *P* < 0.05 for ConA versus ConA + shikonin).

We investigated the expression of Bcl-2, Bax, caspase 9, LC3, Beclin-1, and P62. Bcl-2, Bax, and caspase 9 are markers of apoptosis. Beclin-1, LC3, and P62 play important roles in the process of autophagy. We used real-time PCR and western blot technologies to detect the expression of apoptosis and autophagy markers at the mRNA and protein levels, as shown in Figures 4(a) and 4(b). Bax and caspase 9, the proapoptotic proteins, were significantly upregulated in the ConA group and downregulated in the shikonin pretreatment groups at all the three time points. Bcl-2, an antiapoptotic marker, was downregulated in the ConA group and upregulated in the shikonin treatment groups. The expression of LC3, Beclin-1, and P62 was inhibited with increasing drug doses, with the highest level in the ConA group.  Figure 4(c) shows that the results of immunohistochemistry were consistent with those of western blotting. In conclusion, these results provided strong evidence that shikonin can attenuate hepatocyte apoptosis and autophagy and protect the liver tissue from pathological damage in ConA-induced autoimmune hepatitis.

### 3.5. Shikonin Inhibited the JNK/p-JNK Signaling Pathway in ConA-Induced Hepatitis

**Figure 5:**
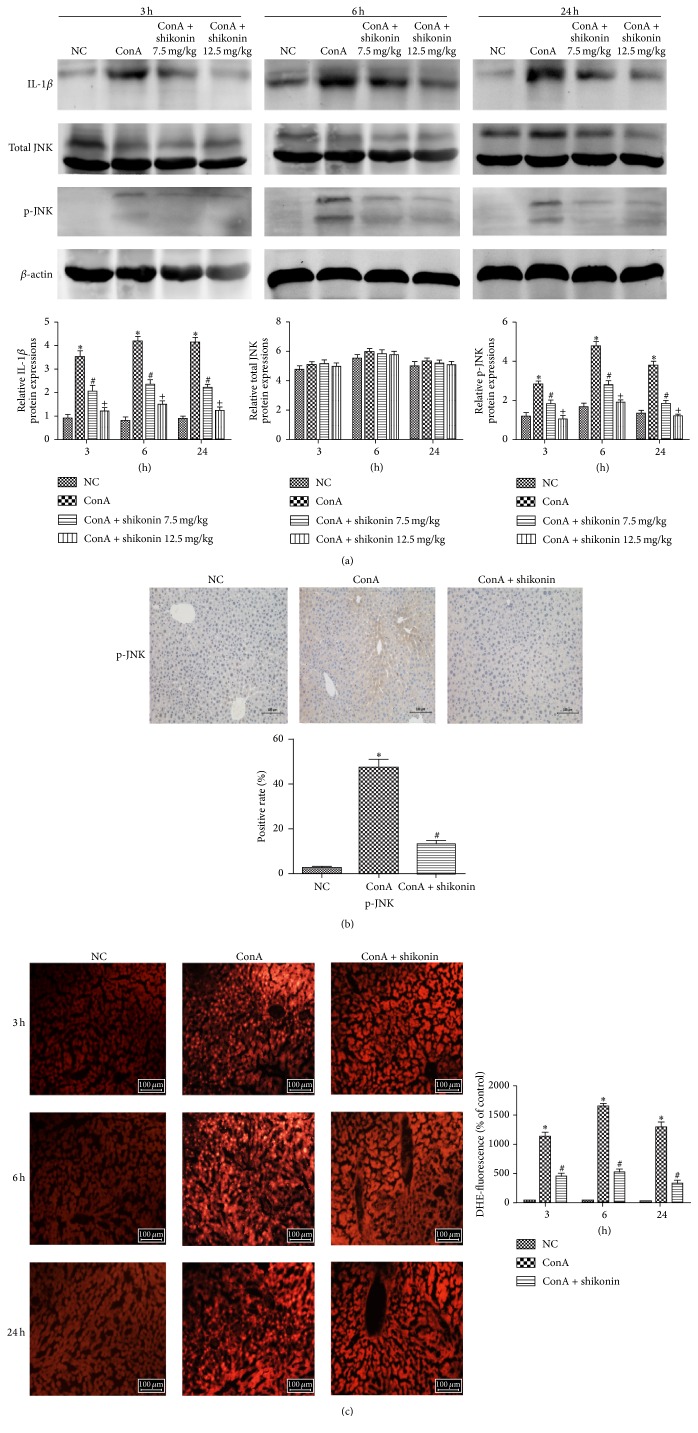
Effects of shikonin on the regulation of the IL-1*β*/JNK/p-JNK pathway in mice with ConA-induced acute hepatitis. (a) The levels of proteins IL-1*β*, total JNK, and p-JNK in liver tissue are shown as western blot bands and the gray values were calculated (*n* = 8, ^*∗*^ *P* < 0.05 for NC versus ConA, ^#^ *P* < 0.05 for ConA + shikonin (7.5) versus ConA, and ^+^ *P* < 0.05 for ConA + shikonin (12.5) versus ConA). (b) The expression of p-JNK in hepatic tissues was determined with immunohistochemistry at 6 h (original magnification, ×200). The ratio of brown area to total area was analyzed with Image-Pro Plus (v 6.0) (*n* = 8, ^*∗*^ *P* < 0.05 for NC versus ConA and ^#^ *P* < 0.05 for ConA versus ConA + shikonin). (c) The generation of ROS was detected with ROS Fluorescent Probe-DHE. And ROS were measured and analyzed in three random vision fields by Image-Pro Plus 6.0 (*n* = 8, ^*∗*^ *P* < 0.05 for NC versus ConA and ^#^ *P* < 0.05 for ConA + shikonin versus ConA).

We showed that shikonin can attenuate autoimmune hepatitis caused by ConA through the inhibition of the release of proinflammatory cytokines, such as IL-1*β*, TNF-*α*, and IFN-*γ*. However, the underlying mechanism remains unclear. Evidence indicates that the JNK/p-JNK pathway is closely associated with inflammatory responses and the progression of apoptosis and autophagy. Therefore, we examined whether shikonin could protect liver tissues by suppressing the JNK/p-JNK pathway in ConA-induced hepatitis. Western blot analysis was used to determine the protein levels of total JNK and the phosphorylation of JNK. The protein levels of total JNK did not differ between the four groups. However, the level of phosphorylated JNK was significantly increased in the ConA-treated group and clearly decreased in the shikonin-pretreated groups at all the three time points (Figure 5(a)). Immunohistochemical staining was used to detect the expression of p-JNK, as shown in Figure 5(b). The results of western blotting and immunohistochemical staining suggested that shikonin treatment can ameliorate liver injury in ConA-induced hepatitis in part through the JNK/p-JNK pathway. There are many factors related to the phosphorylation of JNK. Previous studies have shown that IL-1*β* plays a crucial role in ConA-induced hepatitis and was suggested to play a role in the activation of JNK.  Figure 5 shows that the protein level of IL-1*β* was consistent with the changes of p-JNK. We concluded that shikonin could downregulate the JNK pathway by inhibiting the expression of IL-1*β*.

## 4. Discussion

Autoimmune hepatitis is an inflammatory disease and its incidence has been increasing, which has resulted in an important global burden and poses a serious risk to human lives. However, there are no effective treatment measures, underscoring the need to identify effective therapy options. Shikonin, a promising anti-inflammatory drug, has attracted the attention of scientists worldwide.

The ConA model is a representative and easily built model of autoimmune hepatitis. Several studies show that the release of inflammatory cytokines is involved in autoimmune hepatitis, including IL-1*β*, TNF-*α*, IFN-*γ*, and IL-6, giving rise to the development of liver injury [7, 26]. IL-1*β*, which is secreted by activated macrophages, plays a significant role in the necrosis of liver tissues [27]. Several studies have shown that shikonin exerts anti-inflammatory effects by downregulating the expression of the inflammatory cytokine IL-1*β* in animal models [24, 28]. However, the mechanism of shikonin in concanavalin A-induced autoimmune hepatitis remains largely uncertain and requires further investigation.

Here, we established a model of ConA-induced acute hepatitis to investigate the mechanism underlying the anti-inflammatory effect of shikonin. Our results showed that shikonin pretreatment has a protective effect on ConA-induced liver injury, as shown by serum liver enzymes levels, the release of inflammatory cytokines, and pathological changes. The levels of serum AST and ALT and the range of necrosis of liver tissues on biopsy were markedly reduced by both doses of shikonin (7.5 mg/kg and 12.5 mg/kg), particularly in the high dose group. PCR and western blot technologies were used to detect the expression of inflammatory factors. Our results showed that pretreatment with shikonin suppressed the release of the inflammatory cytokines TNF-*α* and IFN-*γ* and most significantly IL-1*β*. Therefore, we examined shikonin could prevent liver tissue from injury caused by ConA by inhibiting the expression of IL-1*β* and the phosphorylation of JNK [29, 30].

JNK, one of the members of the family of mitogen-activated protein kinases (MAPKs), is activated by many types of factors, such as immune responses, cell stress, and especially the inflammatory cytokine IL-1*β* [31, 32]. The active form of JNK is p-JNK, which is closely associated with apoptosis induced by IL-1*β* [29]. The phosphorylation of JNK is related to many diseases. Ferdaoussi et al. reported that the apoptosis of pancreatic beta-cells induced by IL-1*β* occurs mainly via the activation of the JNK pathway [29]. Nikulina et al. revealed that the cell-permeable peptide inhibitor of JNK (JNKI1) could effectively protect beta-cells from death induced by IL-1*β* [33]. Kim et al. found that the JNK pathway is closely associated with IL-1*β* production in Alzheimer's disease [30].

ConA administration induced the phosphorylation of JNK to generate p-JNK, which was translocated from the cytoplasm to the mitochondrial membrane or cell nucleus causing liver injury. Lee et al. found that ellagic acid (EA) pretreatment significantly attenuated liver injury in ConA-induced hepatitis through the phosphorylation of JNK [34]. A recent study showed that astaxanthin protects liver tissues in a model of ConA-induced immune hepatitis via the JNK/p-JNK pathway [15]. However, the underlying pathophysiological mechanism of shikonin is not clear. We examined whether shikonin protected liver tissues in ConA-induced hepatitis via the inhibition of the JNK/p-JNK signaling pathway. We used western blotting and immunohistochemistry to examine the expression of p-JNK. Our results showed that ConA enhanced the expression of p-JNK, and this was significantly decreased by pretreatment with shikonin, indicating that shikonin attenuated liver injury caused by ConA by reducing the phosphorylation of JNK.

**Figure 6:**
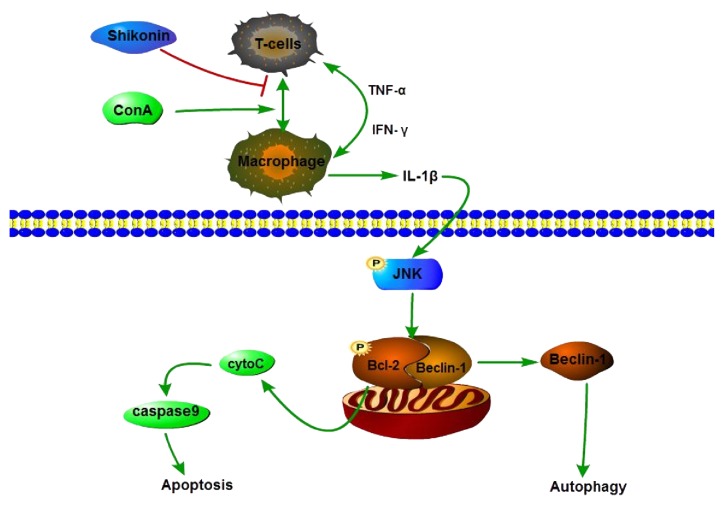


To investigate how shikonin reduced liver injury by modulating the phosphorylation of JNK, we detected the expression of Bcl-2, Bax, and caspase 9. Bcl-2 and Bax belong to the Bcl-2 family, which is an antiapoptotic protein, while Bax represents a proapoptotic protein, and the balance of Bcl-2 and Bax determines cell survival or cell apoptosis [35] (Figure 6). The phosphorylation of JNK results in the inactivation and phosphorylation of Bcl-2, leading to the release of cytochrome C and the activation of caspase-mediated apoptosis [36]. Our results showed that pretreatment with shikonin could increase the expression of Bcl-2 and reduce the expression of Bax and caspase 9 through the effect of p-JNK. These results indicated that shikonin could attenuate hepatic cell apoptosis in ConA-induced hepatitis through the JNK/p-JNK pathway.

Recent studies showed that Bcl-2 is a central regulator of autophagy and apoptosis and functions by interacting with Beclin-1 [37]. When Bcl-2 is inactivated by the effect of p-JNK, it causes the displacement of Bcl-2 from Beclin-1, thereby triggering autophagy. The induction of autophagy results in the formation of autophagosomes, which can generate several markers of autophagy, including LC3, Beclin-1, and P62. The results of PCR and western blotting demonstrated that shikonin pretreatment decreased the expression of LC3 and Beclin-1 and increased the expression of P62 compared with the ConA-treated group. These results indicated that shikonin could protect liver tissues by inhibiting autophagy.

ConA-induced hepatitis involves many complex and multifactorial mechanisms and these mechanisms need to be investigated further. Moreover, additional studies are needed to examine the protective effects of shikonin on liver injury.

## Figures and Tables

**Table 1:** 

Gene		Primer sequence (5′-3′)
IL-1*β*	Forward	CGATCGCGCAGGGGCTGGGCGG
	Reverse	AGGAACTGACGGTACTGATGGA

TNF-*α*	Forward	CAGGCGGTGCCTATGTCTC
	Reverse	CGATCACCCCGAAGTTCAGTAG

IFN-*γ*	Forward	GCCACGGCACAGTCATTGA
	Reverse	TGCTGATGGCCTGATTGTCTT

Bcl-2	Forward	GCTACCGTCGTCGTGACTTCGC
	Reverse	CCCCACCGAACTCAAAGAAGG

Bax	Forward	AGACAGGGGCCTTTTTGCTAC
	Reverse	AATTCGCCGGAGACACTCG

P62	Forward	GAGGCACCCCGAAACATGG
	Reverse	ACTTATAGCGAGTTCCCACCA

LC3	Forward	GACCGCTGTAAGGAGGTGC
	Reverse	AGAAGCCGAAGGTTTCTTGGG

*β*-actin	Forward	GGCTGTATTCCCCTCCATCG
	Reverse	CCAGTTGGTAACAATGCCATGT
